# Time-resolved evaluation of compound repositioning predictions on a text-mined knowledge network

**DOI:** 10.1186/s12859-019-3297-0

**Published:** 2019-12-11

**Authors:** Michael Mayers, Tong Shu Li, Núria Queralt-Rosinach, Andrew I. Su

**Affiliations:** The Scripps Research Institute, 10550 N Torrey Pines Rd, La Jolla, CA 92037 USA

**Keywords:** Heterogeneous network, Semantic Medline database, Semantic network, Unified medical language system, Drug central, Compound repositioning, Machine learning

## Abstract

**Background:**

Computational compound repositioning has the potential for identifying new uses for existing drugs, and new algorithms and data source aggregation strategies provide ever-improving results via in silico metrics. However, even with these advances, the number of compounds successfully repositioned via computational screening remains low. New strategies for algorithm evaluation that more accurately reflect the repositioning potential of a compound could provide a better target for future optimizations.

**Results:**

Using a text-mined database, we applied a previously described network-based computational repositioning algorithm, yielding strong results via cross-validation, averaging 0.95 AUROC on test-set indications. However, to better approximate a real-world scenario, we built a time-resolved evaluation framework. At various time points, we built networks corresponding to prior knowledge for use as a training set, and then predicted on a test set comprised of indications that were subsequently described. This framework showed a marked reduction in performance, peaking in performance metrics with the 1985 network at an AUROC of .797. Examining performance reductions due to removal of specific types of relationships highlighted the importance of drug-drug and disease-disease similarity metrics. Using data from future timepoints, we demonstrate that further acquisition of these kinds of data may help improve computational results.

**Conclusions:**

Evaluating a repositioning algorithm using indications unknown to input network better tunes its ability to find emerging drug indications, rather than finding those which have been randomly withheld. Focusing efforts on improving algorithmic performance in a time-resolved paradigm may further improve computational repositioning predictions.

## Background

Compound repositioning is the identification and development of new uses for previously existing drugs. Repositioning is an attractive pipeline for drug development primarily due to the reduced pharmaceutical uncertainty and development times when compared to traditional pipelines [1]. While clinical observation and improved understanding of the mechanism of action are the two primary means by which a drug is repositioned, computational repositioning provides a third route to identifying these candidates. This third method has seen much development in the past decade as a way to potentially speed up the drug discovery process. The ultimate goal of computational repositioning is to quickly produce a small number of clinically relevant hits for further investigation. This process is achieved through the identification of features that relate drugs to diseases and utilizes a gold standard of known true drug-treats-disease relationships to train an algorithm to categorize or rank potential drug-disease pairs for treatment probability. While this path can efficiently produce repositioning probabilities for countless drug-disease pairs, identifying and experimentally validating the results of clinical importance can be both costly and challenging [2].

In the last decade, there have been many improvements in approaches and algorithms to identify these candidates [3]. These include an expansion from gene expression-based approaches [4, 5] to include methods based on knowledge graphs [6, 7]. Coupled with the advancements in machine learning, the number of different methods for producing repurposing predictions has quickly increased, each showing marked improvements on their ability to accurately predict candidates. One common result in these knowledge-based approaches is that drug-drug and disease-disease similarity, when combined with drug-disease associations, provide the important information for generating a learning model [6, 8, 9]. Many different metrics can be used to express these similarities, like structural motifs in the case of drugs, or phenotypes in the case of diseases. However, as good as these algorithms have become at providing repurposing candidates from a list of known indications, the majority of computational repositioning projects do not continue beyond the in vitro studies [10].

One recent effort in computational repositioning, Himmelstein et al.’s Rephetio project [11] used a heterogeneous network (hetnet) to describe drug-disease relationships in a variety of ways. A hetnet is a network where nodes and edges of more than one type, allowing for multiple edges between any two nodes, each with different semantic meaning. For example, in a simple hetnet with three node types (or metanodes) of Drug, Gene and Disease hetnet, one relationship or metaedge may be a Drug - Inhibits - Gene edge, while another may be a Drug - Activates - Gene Edge. This Rephetio study compiled several different highly curated data sources to generate a hetnet of 11 metanodes and 24 metaedges and produced repositioning predictions by extracting counts of various metapaths between drug-disease pairs, where a metapath is defined by the concept and relationship types in the knowledge graph that join the drug and disease. These metapaths counts were then used as numerical features in a machine learning model, achieving excellent performance results. Whether this learning model that utilizes network structure as features can achieve similar results with a less well-curated network remains an open question.

Progress in the field of natural language processing (NLP) has led to the ability to generate large biomedical knowledge bases through computational text-mining [12, 13]. This method can produce large amounts of data rather quickly, which when coupled with semantic typing of concepts and relations, produces a massive datasource that can quickly be represented in a hetnet structure.

In this work, we evaluated the utility of text-mined networks for use in computational compound repositioning, by utilizing the Semantic MEDLINE Database (SemMedDB) [14] as an NLP-derived knowledge network, and the Rephetio algorithm for producing predictions. We evaluated the performance of this data source when trained with a gold standard of indications taken from DrugCentral [15] and tested via cross-validation. We then propose a new framework for evaluating repurposing algorithms in a time-dependent manner. By utilizing one of the unique features of SemMedDB, a PubMed Identification number (PMID) documented for every edge in the network, multiple networks were produced in a time-resolved fashion, each with data originating on or before a certain date, representing the current state of knowledge at that date. These networks were then evaluated in the context of computational repositioning via training on indications known during the time period of the given network and tested on indications approved after the network, a paradigm that more closely resembles the real-world problem addressed by computational repositioning than a cross-validation. Finally, we analyzed these results to identify the types of data most important to producing accurate predictions and tested the predictive utility of supplementing a past network with future knowledge of these important types.

## Methods

### Initial SemMedDB network generation

The SemMedDB SQL dump Version 31R, processed through June 30, 2018, was downloaded (https://skr3.nlm.nih.gov/SemMedDB/download/download.html) and converted into a csv. Using Python scripts (https://github.com/mmayers12/semmed/tree/master/prepare), corrupted lines were removed, and lines were normalized to a single subject-predicate-object triple per line. Identifiers in this ‘clean’ database were retained in their original Unified Medical Language System (UMLS) space, using the UMLS Concept Unique Identifier (CUI) as the primary ID. This ‘clean’ database was then further processed into a heterogeneous network (hetnet) compatible with the hetnet package, hetio (https://github.com/hetio/hetio) a prerequisite for the rephetio machine learning pipeline [16].

The high computational complexity of feature extraction for this algorithm and non-linear relationship between feature number and unique metaedges necessitated additional processing to reduce complexity. This processing included: using the UMLS Metathesaurus version 2018AA to map terms to other identifier spaces (primarily Medical Subject Headings or MeSH), as MeSH terms tend to be more general than their other counterparts, this mapping functioned to combine granular concepts into more general terms, thus reducing node-count and data-redundancy; combining semantic (edge) types of similar meaning (e.g. between Chemicals & Drugs and Disorders, ‘TREATS’, ‘PREVENTS’, ‘DISRUPTS’, and ‘INHIBITS’ were merged to ‘TREATS’. The full mapping is available here: https://github.com/mmayers12/semmed/blob/master/data/edge_condense_map.csv); filtering out semantic edge types that were sparsely populated (less than 0.1% of the total network); removing the top 100 nodes by degree to eliminate extremely general concepts (e.g., Patients, Cells, Disease, Humans); filtering out edges with less than 2 supporting PMIDs to reduce data noise due to text-mining.

To create time-resolved knowledge networks, a map between PMID and publication year was generated. The primary source for this map was NLM - Baseline Repository (ftp://ftp.ncbi.nlm.nih.gov/pubmed/baseline/), however this was not exhaustive list of PMIDs contained in SemMedDB, so three other data sources were used to create as complete a map as possible: Pubmed Central (ftp://ftp.ncbi.nlm.nih.gov/pub/pmc/), Euro PMC (http://europepmc.org/ftp/pmclitemetadata/), and EBI’s API (https://europepmc.org/RestfulWebService). Networks were generated at 5-year intervals starting at the year 1950 continuing to present day. The PMID with the earliest publication year for a given edge was used for that edge.

### Gold standard generation

The PostgreSQL dump of DrugCentral dated 2018-06-21 was downloaded for use as the gold standard of known drug-disease indications. The following tables were extracted for use throughout the analysis pipeline: *omap_relationship*, containing the indications; *identifier*, with maps from internal IDs to other systems including UMLS and MeSH; *approval*, containing approval dates from worldwide medical agencies; *synonyms*, containing drug names. Both DrugCentral’s and UMLS’s cross-references to MeSH were used to map DrugCentral internal structure IDs to SemMedDB, ensuring maximum overlap. Disease concepts contained both MeSH and Systematized Nomenclature of Medicine (SNOMED) identifiers that could be mapped to SemMedDB via UMLS cross-references. Some diseases could not be mapped to UMLS, primarily due to the specific nature of the condition, and were discarded. Unmappable conditions included ‘Uremic Bleeding Tendency’, ‘Tonic-Clonic Epilepsy Treatment Adjunct’, and ‘Prevention of Stress Ulcer.’ To further merge highly related diseases beyond the mapping UMLS terms to MeSH, diseases were mapped up the Disease Ontology hierarchy to published slim subsets (https://github.com/DiseaseOntology/HumanDiseaseOntology/tree/master/src/ontology/subsets)(https://github.com/dhimmel/disease-ontology) resulting in a more general disease concept for each treated disease. For example, ‘Vasomotor rhinitis,’ ‘Allergic rhinitis’, ‘Perennial allergic rhinitis’, and ‘Seasonal allergic rhinitis,’ were merged through these steps into the single concept ‘Allergic rhinitis.’ For time-resolved analysis, the first approval year for a drug in an indication, provided by DrugCentral, was taken as a proxy for the date of the indication.

### Repurposing algorithm

A customized version of the PathPredict algorithm [17] utilized in the Repehtio repurposing project [11] was adapted for producing repurposing predictions on the SemMedDB hetnet. This algorithm utilizes Degree Weighted Path Counts (DWPC) as the primary feature for machine learning [16]. These features are based on the various metapaths that connect the source and target node types (in this case Chemicals & Drugs, and Disorders). To aid in the speed of feature extraction, we built a framework (https://github.com/mmayers12/hetnet_ml) based on multiplication of Degree-Weighted adjacency matrices to extract path-counts quickly. The extracted features were then scaled and standardized according to the Rephetio framework. Finally, an ElasticNet regularized logistic regression was performed using the python wrapper (https://github.com/civisanalytics/python-glmnet) for the Fortran library used in the R package glmnet [18]. Two hyperparameters were tuned via grid search, the ElasticNet mixing parameter (*α*) and the DWPC damping exponent (*w*) and once chosen left constant throughout all future runs.

To evaluate the model, the DrugCentral gold standard was partitioned by indication into 5 equal partitions. One-fifth of the indications were withheld during training, and negative training examples were sampled at a rate of ten times the number of positives from the set of non-positive drug-disease pairs. The corresponding TREATS edges for holdout indications were removed from the hetnet before feature extraction in an attempt to limit the model’s ability to learn directly from those edges. The five-fold cross-validations were performed a total of ten times, each with a different random partitioning.

### Time-restricted learning models

The models for the time-resolved networks were trained using the positive gold-standard indications where drug was approved in the years prior to and including the year of the network. Negatives were selected randomly from the pool of non-positive drug-disease pairs at a ratio of 10:1 of the total number of positives, prior and future. Negatives were then split 80:20 into training and validation sets. After training, the models were then tested on a combination of the positive indications dated after the year of the network and the 20% of the selected negatives. Breakdown of the number of positive and negative examples in each split is detailed in Additional file 1: **Table S1**.

To combine the results of all of the models across the all network years, the prediction probability for each model was first converted to z-score. This allowed for a cross model comparison of the results. The standardized probabilities for gold-standard drug-disease indications were then grouped according to the difference in years between the network version and the approval year of the drug in the indication. This grouping allowed for the generation of performance metrics for a relative drug approval year. Negative examples were again randomly selected at a ratio of 10:1, across all models. Area under the receiver operator characteristic (AUROC) and precision recall curves (AUPRC) were then calculated for each of the time differences from negative 20 to positive 20 years.

### Feature performance analyses

To test the relative importance of each edge type to the model, one of the better performing networks on future indications, 1985, was chosen as a baseline. We performed a ‘dropout’ analysis in which edge instances were removed randomly from the network at rates of 25, 50, 75, and 100% before running the machine learning pipeline. Randomly dropping a fraction of the edges may result in edges more informative to the model may be removed, while other less-import remain, or vise-versa. To account for this, five replicates were run at each of the dropout rates, with random seeds used to select the edges to drop. Performance metrics AUROC and AUPRC of these different dropout results were then compared to the baseline 1985 network model result.

For the edge replacement analysis, the 1985 network was taken as a baseline. Edge instances of a given type were, type by type, replaced with those from the networks of other years starting with 1950 and continuing to present. This produced 15 models for each of the 30 edge types, one for each network year per edge type. For example, for the TREATS edge, all values from the 1985 network were removed and replaced with TREATS edges from the 1950 network and predictions were made, then the TREATS edges were replaced with those from the 1955 network, and so-forth. AUROC and AUPRC results from these modified networks were compared to that of the base 1985 network.

## Results

### 5-fold cross-validation on text-mined data

**Fig. 1:**
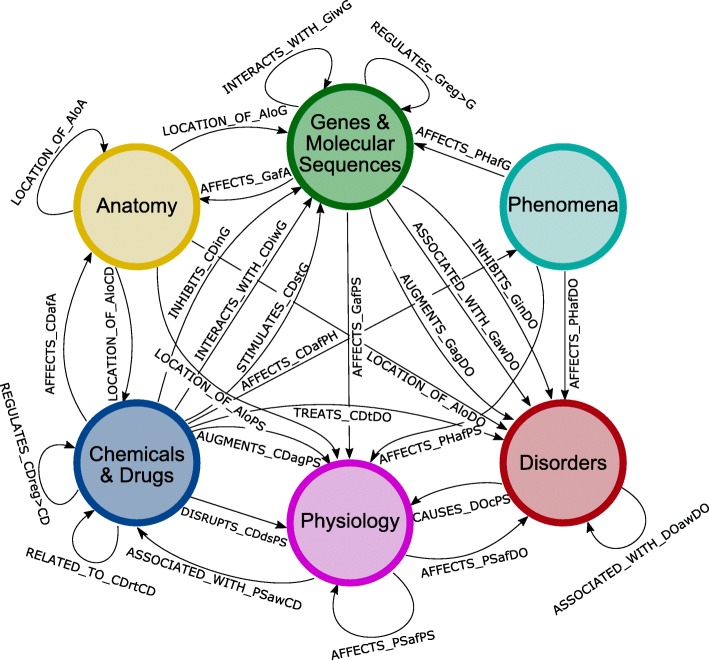
The metagraph SemMedDB hetnet data model. This graph details the 6 node types and 30 edge types present in this network

A hetnet comprised of biomedical knowledge was built from SemMedDB, a database containing subject, predicate, object triples that were text-mined from PubMed abstracts. The initial SemMedDB data dump contained 21,416,739 unique subject predicate object triples (graph edges) and 263,692 unique concepts (nodes). After data processing steps (see methods) the final network contained 78,400 nodes and 2,470,050 edges. These concepts were classified into 6 different types derived from the UMLS semantic groups – ‘Chemicals & Drugs’, ‘Disorders’, ‘Genes & Molecular Sequences’, ‘Anatomy’, ‘Physiology’, and ‘Phenomena’. The relationships between the nodes were also classified as one of 30 different edge types, comprised of both a semantic relation and the source and target node types. For example, the relation ‘AFFECTS’ between nodes of type ‘Chemicals & Drugs’ and ‘Anatomy’ is distinct from the relationship ‘AFFECTS’ between nodes of type ‘Chemicals & Drugs’ and ‘Physiology’. In labeling these relations, the node abbreviations are appended to the semantic relation to explicitly differentiate the edge types, e.g. the above examples the labels are ‘AFFECTS_CDafA’ and ‘AFFECTS_CDafPH’ respectively (Table 1, and Fig. 1). To train a learning model for compound repurposing, a gold standard of high quality and reliability containing drug-disease indications is required. We used the open source drug database, DrugCentral, as the source for our gold standard, as this database contains a relatively complete, curated list of known indications, with a total of 10,938 unique drug-disease pairs. In mapping these drug and disease concepts to those found in SemMedDB, 2489 indications were lost due a lack of any identifier in DrugCentral (see methods for examples). Another 1396 lost due to an inability to map either the drug or the disease to a UMLS concept found in SemMedDB. Further reductions came due to the merging of highly related disease concepts, resulting in 5337 unique indications that could be used as true-positives for training and testing purposes.

**Table 1:** Top 10 Edge Types by Instance Number

Subject Node Type	Predicate	Object Node Type	Edge Abbreviation	Count
Anatomy	LOCATION_OF	Chemicals & Drugs	AloCD	380,422
Chemicals & Drugs	REGULATES	Chemicals & Drugs	CDreg>CD	214,912
Chemicals & Drugs	INTERACTS_WITH	Genes & Molecular Sequences	CDiwG	183,016
Anatomy	LOCATION_OF	Disorders	AloDO	182,373
Anatomy	LOCATION_OF	Genes & Molecular Sequences	AloG	174,246
Chemicals & Drugs	TREATS	Disorders	CDtDO	172,384
Disorders	ASSOCIATED_WITH	Disorders	DOawDO	169,075
Anatomy	LOCATION_OF	Anatomy	AloA	98,472
Chemicals & Drugs	STIMULATES	Genes & Molecular Sequences	CDstG	93,343
Chemicals & Drugs	AFFECTS	Anatomy	CDafA	92,126

**Fig. 2:**
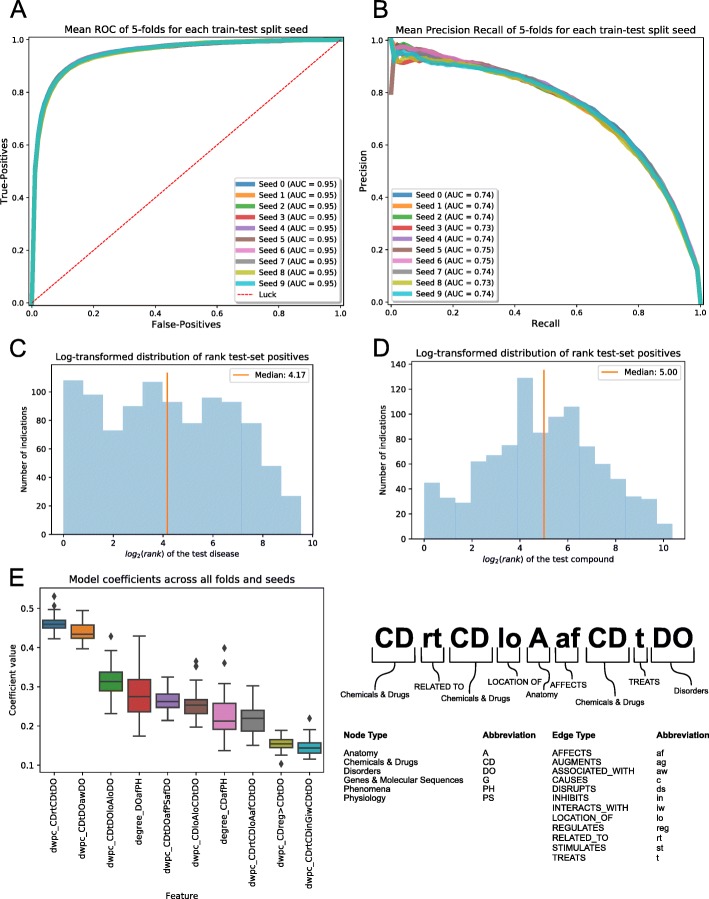
5-fold cross validation results for SemMedDB network using DrugCentral gold standard. **a)** Receiver-Operator Characteristic curve displaying the mean result across 5-folds. Ten different seed values for randomly splitting indications in 5 are compared showing very little variation. **b)** Precision-Recall curve for the mean result across 5-folds, with ten different split seeds displayed. **c)** Histogram of log_2_ transformed rank of true positive disease for a given test-set positive drug, taken from a representative fold and seed of the cross-validation. If a drug treats multiple diseases, the ranks of all diseases treated in the test-set indications are shown. **d)** Histogram of log_2_ transformed rank of true positive drug for a given test-set disease, chosen from same fold and seed as C. If a disease is treated by multiple drugs in the test-set indications, all ranks are included. **e)** (left) Boxplot of 10 largest model coefficients in selected features across all folds and seeds. (right) Breakdown of metapath abbreviations. Node abbreviations appear in capital letters while edge abbreviations appear lower case

After preparation of the hetnet and the gold standard, the utility of this text-mined knowledge base for the prediction of novel drug-disease indications was examined using a modified version of the PathPredict algorithm, utilized by Himmelstein et al. in the Rephetio drug repurposing project [11]. This paradigm utilizes metapaths, or the ways that the node types (e.g. Gene & Molecular Sequences) and edge types (e.g. INTERACTS_WITH) combine to produce a path from Drug to Disease, as the primary features for machine learning. Counts of each metapath between Drug and Disease, then are weighted by the degree of the nodes within each path, producing the degree weighted path count (DWPC) metric as the primary features for training the classifier [16]. The remaining features, while comparatively small, are derived from the simple degree values of each edge type for the drug node and the disease node in given drug-disease pair. The models produced during 5-fold cross validation showed excellent results, with an average area under the receiver operator characteristic (AUROC) of 0.95 and average precision (AUPRC) of 0.74 (Fig. 2a and b). These results are consistent with a very accurate classifier, and comparable to results seen in similar computational repositioning studies [6, 9, 11]. To further evaluate the accuracy of these predictions, the prediction rankings of validation set indications were examined for given drugs and diseases (Fig. 2c and d). The median value for the rank of a positive disease, given a test-set positive drug was 18 out of 740 total diseases. Similarly, when examining the test-set positive diseases, the median rank for a positive drug was 32 out of a possible 1330 examined compounds.

The ElasticNet logistic regression in this analysis used feature selection to reduce the risk of overfitting with a highly complex model. In comparing the models, there was a fairly consistent selection of short metapaths with only two edges that include important drug-drug or disease-disease similarity measures (Fig. 2e). These include two related drugs, one of which treats a disease (dwpc_CDrtCDtDO), or two associated diseases, one of which has a known drug treatment (dwpc_CDtDOawDO). However, other metapaths of length 3 which encapsulated drug-drug or disease-disease similarities were also highly ranked. This includes two drugs that co-localize to a given anatomical structure (dwpc_CDloAloCDtDO), two diseases that present in the same anatomical structure (dwpc_CDtDOloAloDO), or diseases that affect similar phenomena (dwpc_CDtDOafPHafDO). In this case anatomical structures could include body regions, organs, cell types or components, or tissues, while phenomena include biological functions, processes, or environmental effects. It is important to again note that these ‘similarity measures’ are purely derived from text-mined relations.

While these results indicate a fairly accurate classifier in this synthetic setting, the paradigm under which they are trained and tested is not necessarily optimal for finding novel drug-disease indications. A cross-validation framework essentially optimizes finding a subset of indication data that has been *randomly* removed from a training set. However, prediction accuracy on randomly removed indications does not necessarily extrapolate to prospective prediction of new drug repurposing candidates. Framing the evaluation framework instead as one of future prediction based on past examples may be more informative. For example, the question ‘given today’s state of biomedical knowledge, can future indications be predicted?’ may more closely reflect the problem being addressed in drug repositioning. The best way to address this question would be to perform the predictions in a time-resolved fashion, training on contemporary data and then evaluating the model’s performance on an indication set from the future.

### Building time-resolved networks

To facilitate a time-resolved analysis, both the knowledge base data and the training data need to be mapped to a particular time point. Each triple in SemMedDB is annotated with a PMID, indicating source abstract of this text-mined data. Using the PMID, each triple, corresponding to an edge in the final network, can be mapped to a specific date of publication. The DrugCentral database also includes approval dates from several international medical agencies for the majority of the drugs. By filtering the edges in the network by date, an approximate map of the biomedical knowledge of a given time period can be produced. Therefore, we generated multiple networks, each representing distinct time-points. We then applied the machine learning pipeline to each of these networks to evaluate the expected performance on future drug-disease indications. Combining these sources of time-points for the network serves to replicate the paradigm of training a machine learning model on the current state of biomedical knowledge, evaluating its ability to predict what indications are likely to be found useful in the future.

**Fig. 3:**
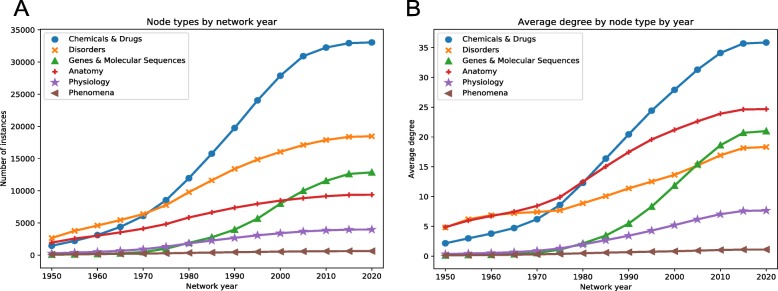
Time-resolved network build results. **a)** Number of nodes of a given type by network year. **b)** Average node degree for each node type across all network years

Knowledge networks were built in a time-resolved fashion for each year, starting with 1950 and continuing until the present. This was accomplished by removing edges with their earliest supporting PMID dated after the desired year of the network. If either a drug or a disease from a known gold standard indication was no longer connected to any other concept in the network, the indication was also removed from the training and testing set for that network year. For example, olprinone, a cardiac stimulant for approved for acute cardiac failure in 1996, was first described in literature in 1989, as stated in SemMedDB. This description was represented hetnet by the edge: olprinone - AUGMENTS_CDagPS - Myocardial Contraction. Because olprinone does not show up in networks before 1989, it is not available for selection in training or validation sets in these network years. Examining the trends of the networks constructed for the various timepoints, the number of nodes and edges always increased, but edges increased more quickly with later timepoints producing a more connected network than earlier (Fig. 3a and b).

The number of indications that could be mapped to a given network year increased quickly at first but rose much more slowly in the later years of the network, even though the total number of concepts in the network continued to increase. For the majority of the years of the network, the split between current and future indications remained at a ratio of around 80% current and 20%, ideal for a training and testing split. However, after the year 2000, the number of mappable future indications continued to diminish year after year, reducing the validation set size for these years (Additional file 1: **Fig. S1**).

### Machine learning results

**Fig. 4:**
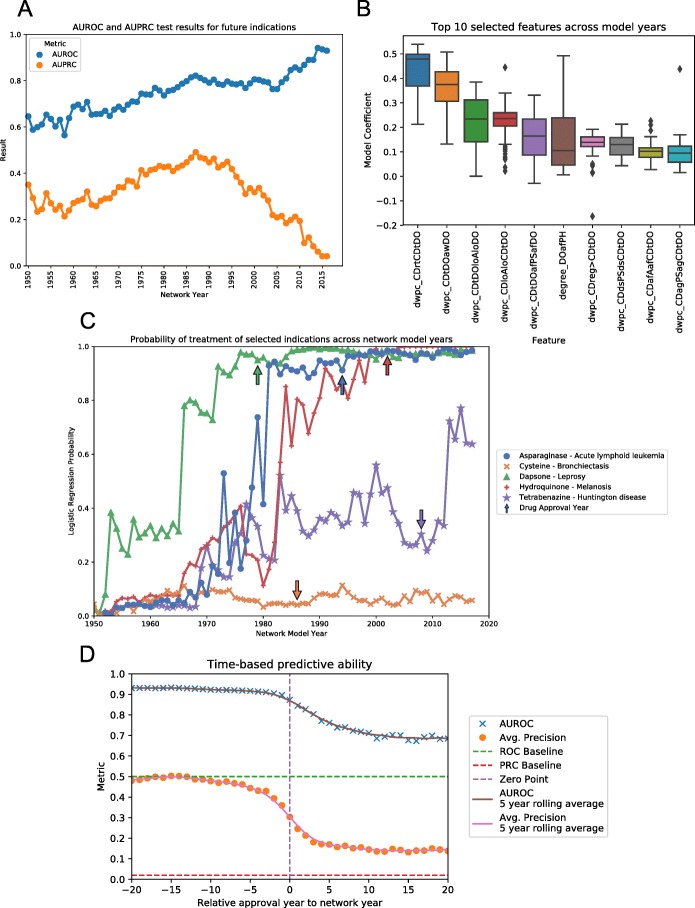
Machine learning results for the time-resolved networks. **a)** Performance metrics for the test-set (future) indications across the different network years. Only drugs approved after the year of the network are included in the test-set, while those approved prior are used for training. **b)** Box plots of the values of the model coefficients across all of the different network years. The top-10 coefficients with largest mean value across all models are shown. **c**) Probabilities of treatment of selected indications for each network model containing both the Drug and Disease concepts. Arrows indicate the year that the drug was first approved for any indication. Points left of the arrow on the graph, the indication was used as part of the validation set, and those to the right, the training set. **d)** AUROC and AUPRC data for indications based on their probabilities, split by the number of years between drug approval date and the year of the network. Values to the left of the Zero Point are indications approved before the network year thus part of the training-set, while those to the right are part of the test-set. Probabilities for all drug-disease pairs were standardized before combining across models. Points are given for each data point, while lines represent a 5-year rolling average of metrics

The performance of each model against a validation set of future indications steadily increased from the earliest time-point until the 1987 network. The AUROC metric saw continual increases over the entirety of the network years, though these increases occurred more slowly after the 1987 network (Fig. 4a). Looking at average precision, this metric peaked at the 1987 timepoint with a value of 0.492, and then fell sharply at 2000 and beyond, likely due to the diminished number of test-set positives. The AUROC of this peak average precision time point of 1985 was 0.822. These peak performance metrics fall far below those found via 5-fold cross-validation indicating an inherent limitation in evaluating models via this paradigm.

Similar to the cross-validation results, the models favored metapaths that represented drug-drug and disease-disease similarity (Fig. 4b). Specifically, the metapaths of type ‘Chemical & Drug - TREATS - Disorder - ASSOCIATED WITH - Disorder’ (dwpc_CDtDOawDO) and ‘Chemical & Drug - RELATED_TO - Chemical & Drug - TREATS - Disorder’ (dwpc_CDrtCDtDO) had the highest weights across almost all models. One difference found from the cross-validation results is the appearance of the `Physiology` metanode in two of the top selected metapaths, one connecting two diseases through common physiology, and one connecting two drugs that both augment a particular physiology. Model complexity was also diminished compared to those seen in during cross-validation, with the majority of models selecting less than 400 features, or 20% of the total available (Additional file 1: Fig. S2).

Finally, one question to explore is whether or not there is a temporal dependence on the ability to predict indications. For example, is there better performance on drugs approved 5 years into the future rather than 20, since one only 5 years pre-approval may already be in the pipeline with some important associations already known in the literature. Looking at selected examples (Fig. 4c), there appeared to be an increase in probability of treatment, as assigned by the Logistic Regression model, before the Drug’s initial approval year. Asparaginase is a treatment for Acute lymphoid leukemia approved by the FDA in 1994. Examining the probability of treatment over time shows very low probability in the early models. However, the probability rises from 1970 to 1980, reaching above 90% by 1981, thirteen years before the drug was approved. This increase in probability before a drugs approval does not hold for all indications. Cysteine, an amino acid that, according to DrugCentral, is used to treat Bronchiectasis. However, this Drug-Disease pair saw no increase in probability of treatment, even after its approval year in 1986, when this indication became a part of the training set. This is likely due to the fact, since cystine is a ubiquitous compound in biomedical literature, the degree of each edge is 2–3 orders of magnitude greater than the average compound for a given edge type. This results in the algorithm treating Cysteine as a hub node, and severely down weighting all DWPC metrics associated with this node, essentially producing a null vector.

To identify whether or not these observations held as a general trend, the results from all network years were combined via z-scores. Grouping indications by approval relative to the year of the network allowed for an AUROC metric to be determined for different timepoints into the future (Fig. 4d). This analysis revealed that there is still a substantial predictive ability for drugs approved up to about 5 years into the future. However, after 5 years, this value quickly drops to a baseline of .70 for the AUROC and .15 for the average precision. These results indicate a temporal dependence on the ability to predict future indications, with the model being fairly inaccurate when looking far into the future.

### Edge dropout confirms importance of drug disease links

**Fig. 5:**
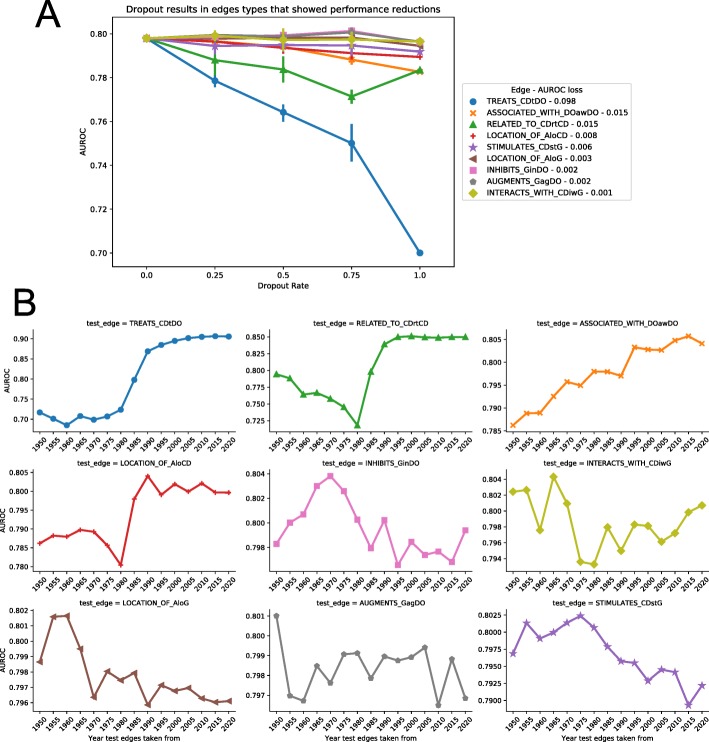
Analysis of edge type importance to the overall model. **a)** Edge dropout analysis showing the reduction in AUROC metric when the edges are dropped out at rates of 25, 50, 75, and 100%. Error bars indicate 95% confidence interval over 5 replicates with different seeds for dropout. The 9 edge types that had the greatest reduction from 0 to 100% dropout are displayed. **b)** Edge replacement analysis showing changes in AUROC when edges are replaced with those of the same type from another year’s network. The top 9 edges that showed greatest loss in performance in the dropout analysis between 0 and 100% dropout are displayed

Many other efforts in computational repositioning have found that emphasis on drug-drug and disease-disease similarity metrics results in accurate predictors [6, 19, 20]. To further investigate the types of information most impactful in improving the final model, an edge dropout analysis was run. The 1985 network was chosen as a base network for this analysis both due to its relatively strong performance on future indications and its centralized time point among all the available networks. By taking each edge type, randomly dropping out edge instances at rates of 25, 50, 75 and 100%, and comparing the resulting models, the relative importance of each edge type within the model could be determined. The edge that was found to have the largest impact on the resulting model was the ‘Chemicals & Drugs - TREATS - Disorders’ edge, reducing the AUROC by .098 (Fig. 5a). This result reinforces the idea that drug-disease links, particularly those with a positive treatment association, are highly predictive in repositioning studies. The drug-drug (‘Chemicals & Drugs - RELATED_TO - Chemicals & Drugs’) and disease-disease (‘Disorders - ASSOCIATED_WITH - Disorders’) similarity edges were the next two most impactful edges on the overall model, both showing decreases of .015 in the AUROC when completely removed. Overall, however most edges showed very little reduction in AUROC, even at 100% dropout rate. This could indicate a redundancy in important connections between drugs and diseases that the model can continue to learn on even when partially removed.

### Time-resolved edge substitution confirms edge importance

While dropout identifies the most important associations between concepts to this predictive model, this does not necessarily confirm that more data of these types will improve the model’s results. To simulate this the impact of the assimilation of new knowledge of a specific type, an edge replacement analysis was performed on the 1985 network. This process allowed for the examination of how accumulating new real-world data of a given type might affect the model. By taking a specific edge type and replacing all the edges of that type with those from the other network years from 1950 to 2015, the potential effect of gathering more data of these specific types over time could be examined. Similar to the dropout analysis, the target edge of ‘Chemicals & Drugs - TREATS - Disorders’ had the greatest effect on the model’s performance, showing an increase of .108 when replaced with the most current version of the edge (Fig. 5b). Similarly, the AUROC showed a large loss of .081 when replaced with values from 1950. The drug-drug and disease-disease similarity edges also showed significant performance increases when replaced with contemporary values, while decreasing performance in performance when replaced with 1950 values. While the three edges that produced the greatest decrease in performance during the dropout analysis also had the biggest benefit when adding future edges, not all behaved in this manner. For example, the edge ‘Anatomy - LOCATION_OF - Chemicals & Drugs’ showed the fourth largest decreases in performance during edge dropout analysis. When using past versions of this edge type with the 1985 network, the performance did have a measurable decrease in AUROC of .012, however current versions of this edge type only improved the score by .002. Conversely, the edge ‘Physiology - AFFECTS - Disorders’ showed little to no performance loss during the dropout analysis and indeed showed little performance change when using past versions of the edge (Additional file 1: **Fig. S3**). However, this edge showed substantial increase of .012 AUROC when using contemporary versions of the edge. Finally, some edge types like ‘Genes & Molecular Sequences - ASSOCIATED WITH - Disorders’ actually performed slightly better with past version or future versions of the edge, when compared 1985 version of the edge, with an increase in AUROC of .004 with contemporary edges and an increase of .011 with edges from 1950 (Additional file 1: **Fig. S4**). This further underscores the idea that a time-resolved analysis provides a more complete picture of the important components to a learning model.

## Discussion

While a text-mined data source, SemMedDB performed very well when using the metapath-based repositioning algorithm from Rephetio and trained and tested against a DrugCentral derived gold standard. However, performing well in a cross-validation does not necessarily lead to a large number of real-world repositioning candidates. This evaluation paradigm essentially trains the learning model to identify indications that are currently known but simply withheld from a dataset. In the real world, the problem solved by computational repositioning is more closely aligned to attempting to predict new indications that are not already known at this current time-point. Our use of time-resolved knowledge networks has allowed us to replicate this paradigm and expose a marked reduction in performance when a model is tested in this fashion. The observed performance reduction combined with the high level of noise in the underlying data source, SemMedDB, contraindicate the utility of performing further validation on the individual repurposing candidates identified in this work.

Time separation is a long-used practice to combat overfitting in data mining [21] and our application of this practice to compound repositioning may help explain some of the discrepancy between model performance and the number of repositioning candidates successfully produced through computational repositioning. We believe that this method for evaluating a repositioning algorithm in a time-resolved fashion may more accurately reflect its ability to find true repurposing candidates. Identifying algorithms that perform well at predicting future indications on the time-resolved networks presented in this paper may yield better results when translating retrospective computational analyses to the prospective hypothesis generation. As these networks are built around text-mined data, predictive performance may be enhanced by utilizing high-confidence, curated, data sources for computational repositioning. The original date of discovery for a given data point has shown itself to be an important piece of metadata in evaluating a predictive model. Ensuring curated data sources are supported by evidence that can be mapped back to an initial date of discovery functions to enhance the utility of the data in predictive models such as these. Finally, this temporal analysis again supports the notion that drug and disease similarity measures as well as direct associations between these concepts are still the most important pieces of data in generating a predictive model. Further enhancing our understanding of mechanistic relationships that these concepts will likely result in further increases to computational repositioning performance.

## Conclusions

Time-resolved evaluation of compound repositioning algorithms provides a better method for determining the ability of an algorithm to find new drug indications than cross-validation alone. Tuning computational repositioning algorithms for better performance in this time-resolved framework could function to improve in silico predictions, hopefully increasing the proportion of hits to pass beyond the in vitro stage.

## Supplementary information

**Additional file 1: **Supplemental Figures **Figure S1.** Number of indications mappable to each network year, split by approval year, with current indications mean those approved up-to and including the network year, and future those approved after the network year. **Figure S2.** Number of features selected in the models for each of the different network years. **Figures S3 & S4.** Edge substitution analysis results for edges AFFECTS_PSafDO and ASSOCIATED_WITH_GawDO respectively. Supplemental Table **Table S1.** Number of training and testing examples in time-resolved analysis

## Data Availability

Data for SemMedDB hetnet building: The SemMedDB database used to build the heterogeneous network analyzed in this study are is available here: https://skr3.nlm.nih.gov/SemMedDB/index.html The UMLS Metathesaurus used for identifier cross-referencing are available https://www.nlm.nih.gov/research/umls/licensedcontent/umlsknowledgesources.html These data are provided by the UMLS Terminology Service, but restrictions apply to the availability of this data, which were used under the UMLS Metathesaurus License. https://www.nlm.nih.gov/databases/umls.html#license_request [14] Data for gold standard: The DrugCentral database used to build the gold standard for this study is freely available from DrugCentral under the CC-BY-SA-4.0 license. http://drugcentral.org/ [15] Source code to download the above datasets and reproduce the analysis found in this current study is available on GitHub in the following repository. https://github.com/mmayers12/semmed Additional Datafiles: The reprocessed version of DrugCentral utilized as training positives in this work, as well as the top 5000 predictions produced by the contemporary network are available on Zenodo under the CC-BY-SA-4.0 license. https://zenodo.org/record/3387731

## Abbreviations

AUPRC: Area Under the Precision Recall Curve (aka average precision)
AUROC: Aera Under the Receiver Operator Curve
DWPC: Degree Weighted Path Count
Hetnet: Heterogeneous network
MeSH: Medical Subject Headings
NLP: Natural Language Processing
PMID: PubMed Identifier
SemMedDB: Semantic Medline Database
UMLS: Unified Medical Language System
