# Supplementary material for: Time-resolved evaluation of compound repositioning predictions on a text-mined knowledge network
Source: BMC Bioinformatics. 2019 Dec 11;20:653. doi: 10.1186/s12859-019-3297-0 (PMC6907279; doi:10.1186/s12859-019-3297-0)
Supplement: Supplementary file 1 — Additional file 1: Supplemental Figures Figure S1. Number of indications mappable to each network year, split by approval year, with current indications mean those approved up-to and including the network year, and future those approved after the network year. Figure S2. Number of features selected in the models for each of the different network years. Figures S3 & S4. Edge substitution analysis results for edges AFFECTS_PSafDO and ASSOCIATED_WITH_GawDO respectively. Supplemental Table Table S1. Number of training and testing examples in time-resolved analysis [file 12859_2019_3297_MOESM1_ESM.docx]

**Supplemental Figures**


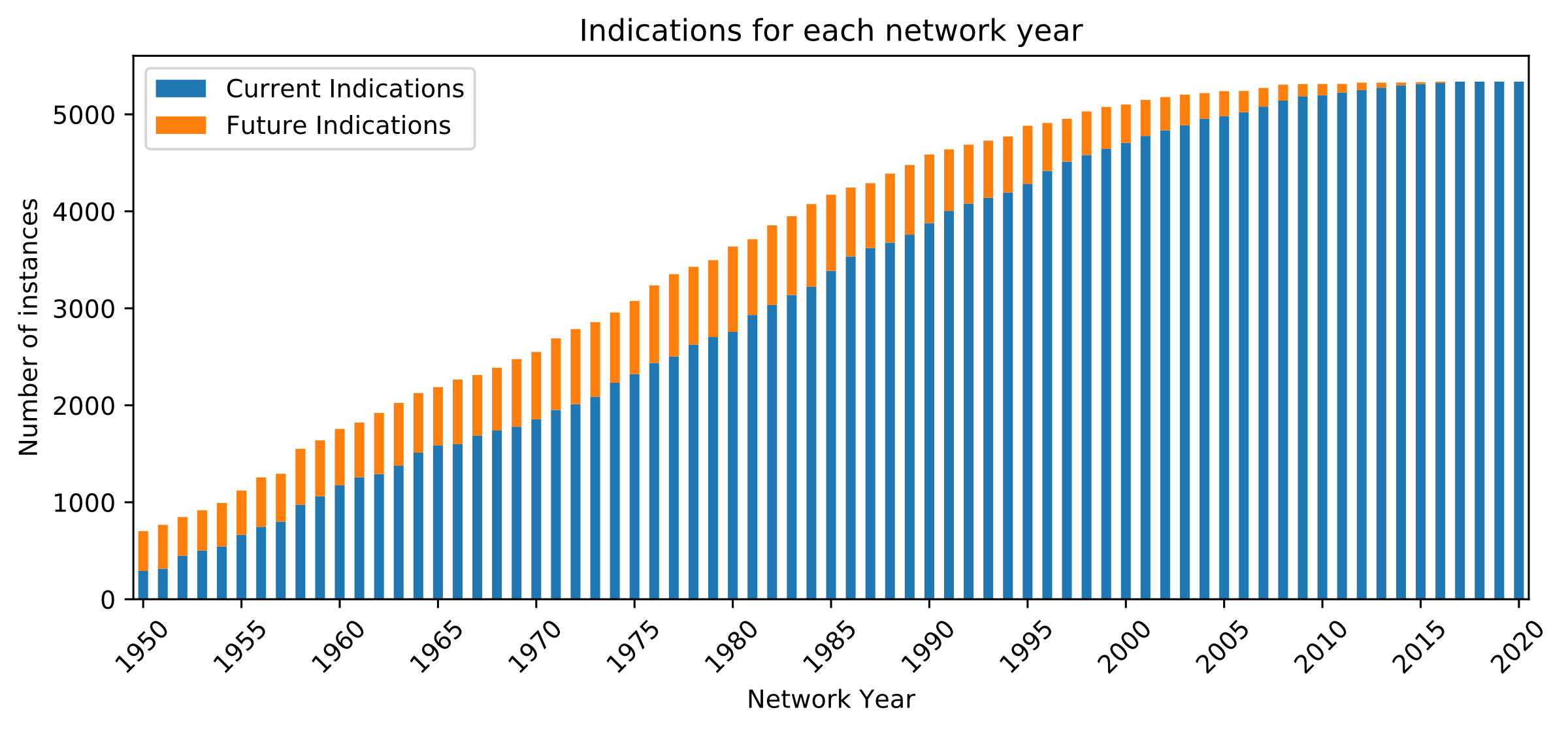


**Supplemental Figure S1)** Number of indications mappable to each network year, split by approval year, with current indications mean those approved up-to and including the network year, and future those approved after the network year.


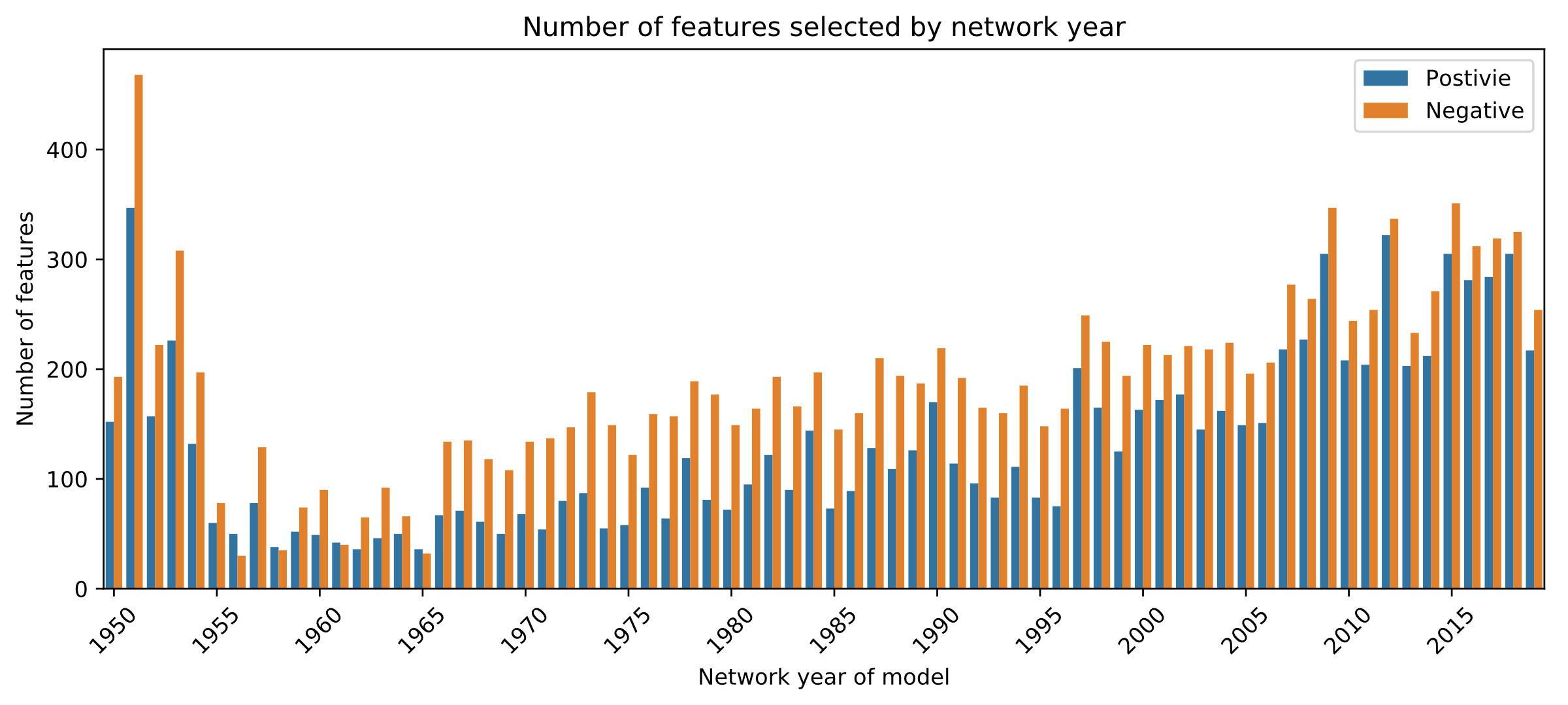


**Supplemental Figure S2)** Number of features selected in the models for each of the different network years.


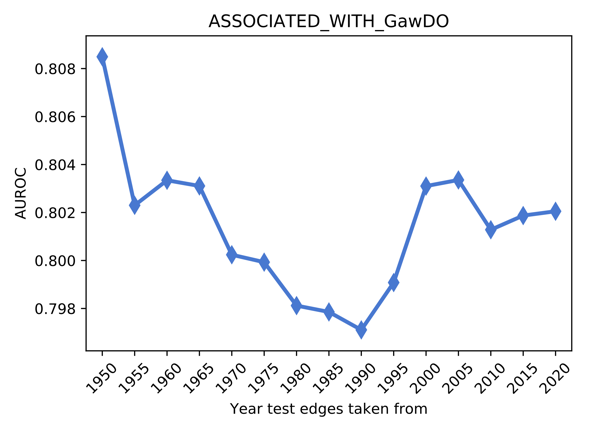

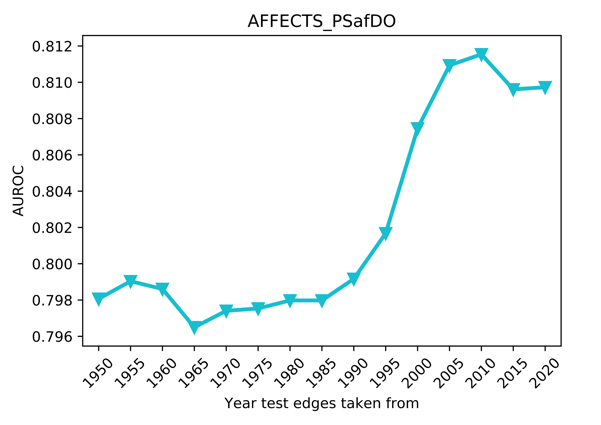


**Supplemental Figures S3 & S4)** Edge substitution analysis results for edges AFFECTS_PSafDO and ASSOCIATED_WITH_GawDO respectively.

**Supplemental Tables**

Supplemental Table S1: Number of training and testing examples in time-resolved analysis

| Network Year | Training Positives | Testing Positives | Total Positives | Training Negatives | Testing Negatives | Total Negatives |
| --- | --- | --- | --- | --- | --- | --- |
| 1950 | 274 | 321 | 595 | 4760 | 1190 | 5950 |
| 1951 | 296 | 341 | 637 | 5096 | 1274 | 6370 |
| 1952 | 424 | 286 | 710 | 5680 | 1420 | 7100 |
| 1953 | 471 | 301 | 772 | 6176 | 1544 | 7720 |
| 1954 | 512 | 332 | 844 | 6752 | 1688 | 8440 |
| 1955 | 627 | 338 | 965 | 7720 | 1930 | 9650 |
| 1956 | 665 | 386 | 1051 | 8408 | 2102 | 10510 |
| 1957 | 703 | 371 | 1074 | 8592 | 2148 | 10740 |
| 1958 | 869 | 449 | 1318 | 10544 | 2636 | 13180 |
| 1959 | 948 | 429 | 1377 | 11016 | 2754 | 13770 |
| 1960 | 1099 | 435 | 1534 | 12272 | 3068 | 15340 |
| 1961 | 1132 | 465 | 1597 | 12776 | 3194 | 15970 |
| 1962 | 1220 | 529 | 1749 | 13992 | 3498 | 17490 |
| 1963 | 1289 | 535 | 1824 | 14592 | 3648 | 18240 |
| 1964 | 1398 | 496 | 1894 | 15152 | 3788 | 18940 |
| 1965 | 1470 | 482 | 1952 | 15616 | 3904 | 19520 |
| 1966 | 1498 | 520 | 2018 | 16144 | 4036 | 20180 |
| 1967 | 1579 | 504 | 2083 | 16664 | 4166 | 20830 |
| 1968 | 1644 | 537 | 2181 | 17448 | 4362 | 21810 |
| 1969 | 1665 | 590 | 2255 | 18040 | 4510 | 22550 |
| 1970 | 1726 | 590 | 2316 | 18528 | 4632 | 23160 |
| 1971 | 1822 | 590 | 2412 | 19296 | 4824 | 24120 |
| 1972 | 1884 | 624 | 2508 | 20064 | 5016 | 25080 |
| 1973 | 1956 | 652 | 2608 | 20864 | 5216 | 26080 |
| 1974 | 2106 | 603 | 2709 | 21672 | 5418 | 27090 |
| 1975 | 2189 | 642 | 2831 | 22648 | 5662 | 28310 |
| 1976 | 2289 | 692 | 2981 | 23848 | 5962 | 29810 |
| 1977 | 2363 | 724 | 3087 | 24696 | 6174 | 30870 |
| 1978 | 2455 | 698 | 3153 | 25224 | 6306 | 31530 |
| 1979 | 2532 | 693 | 3225 | 25800 | 6450 | 32250 |
| 1980 | 2604 | 783 | 3387 | 27096 | 6774 | 33870 |
| 1981 | 2742 | 695 | 3437 | 27496 | 6874 | 34370 |
| 1982 | 2848 | 730 | 3578 | 28624 | 7156 | 35780 |
| 1983 | 2939 | 725 | 3664 | 29312 | 7328 | 36640 |
| 1984 | 3018 | 766 | 3784 | 30272 | 7568 | 37840 |
| 1985 | 3140 | 708 | 3848 | 30784 | 7696 | 38480 |
| 1986 | 3272 | 651 | 3923 | 31384 | 7846 | 39230 |
| 1987 | 3387 | 616 | 4003 | 32024 | 8006 | 40030 |
| 1988 | 3440 | 656 | 4096 | 32768 | 8192 | 40960 |
| 1989 | 3497 | 659 | 4156 | 33248 | 8312 | 41560 |
| 1990 | 3602 | 658 | 4260 | 34080 | 8520 | 42600 |
| 1991 | 3707 | 590 | 4297 | 34376 | 8594 | 42970 |
| 1992 | 3788 | 561 | 4349 | 34792 | 8698 | 43490 |
| 1993 | 3864 | 549 | 4413 | 35304 | 8826 | 44130 |
| 1994 | 3925 | 537 | 4462 | 35696 | 8924 | 44620 |
| 1995 | 4005 | 539 | 4544 | 36352 | 9088 | 45440 |
| 1996 | 4141 | 450 | 4591 | 36728 | 9182 | 45910 |
| 1997 | 4215 | 410 | 4625 | 37000 | 9250 | 46250 |
| 1998 | 4280 | 425 | 4705 | 37640 | 9410 | 47050 |
| 1999 | 4343 | 404 | 4747 | 37976 | 9494 | 47470 |
| 2000 | 4413 | 374 | 4787 | 38296 | 9574 | 47870 |
| 2001 | 4475 | 351 | 4826 | 38608 | 9652 | 48260 |
| 2002 | 4532 | 314 | 4846 | 38768 | 9692 | 48460 |
| 2003 | 4580 | 295 | 4875 | 39000 | 9750 | 48750 |
| 2004 | 4643 | 242 | 4885 | 39080 | 9770 | 48850 |
| 2005 | 4674 | 229 | 4903 | 39224 | 9806 | 49030 |
| 2006 | 4715 | 194 | 4909 | 39272 | 9818 | 49090 |
| 2007 | 4745 | 167 | 4912 | 39296 | 9824 | 49120 |
| 2008 | 4772 | 140 | 4912 | 39296 | 9824 | 49120 |
| 2009 | 4814 | 104 | 4918 | 39344 | 9836 | 49180 |
| 2010 | 4826 | 96 | 4922 | 39376 | 9844 | 49220 |
| 2011 | 4855 | 71 | 4926 | 39408 | 9852 | 49260 |
| 2012 | 4879 | 58 | 4937 | 39496 | 9874 | 49370 |
| 2013 | 4896 | 42 | 4938 | 39504 | 9876 | 49380 |
| 2014 | 4916 | 23 | 4939 | 39512 | 9878 | 49390 |
| 2015 | 4928 | 15 | 4943 | 39544 | 9886 | 49430 |
| 2016 | 4930 | 13 | 4943 | 39544 | 9886 | 49430 |
| 2017 | 4943 | 0 | 4943 | 39544 | 9886 | 49430 |
